# Surface-Localized Chemically Modified Reduced Graphene Oxide Nanocomposites as Flexible Conductive Surfaces for Space Applications

**DOI:** 10.1021/acsapm.3c00588

**Published:** 2023-06-29

**Authors:** Emily A. Ryan, Zach D. Seibers, John R. Reynolds, Meisha L. Shofner

**Affiliations:** † School of Materials Science and Engineering, Georgia Institute of Technology, Atlanta, Georgia 30332, United States; ‡ School of Chemistry and Biochemistry, Center for Organic Photonics and Electronics (COPE), Georgia Tech Polymer Network (GTPN), Georgia Institute of Technology, Atlanta, Georgia 30332, United States

**Keywords:** graphene nanocomposite, reduced graphene oxide, electrical conductivity, electrostatic discharge, melt infiltration, space materials

## Abstract

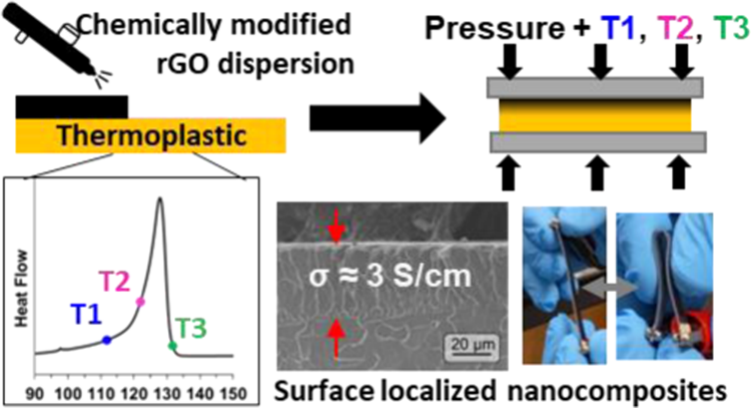

Thermoplastic polymers are a compelling class of materials for emerging space exploration applications due to their wide range of mechanical properties and compatibility with a variety of processing methods, including additive manufacturing. However, despite these benefits, the use of thermoplastic polymers in a set of critical space applications is limited by their low electrical conductivity, which makes them susceptible to static charging and limits their ability to be used as active and passive components in electronic devices, including materials for static charge dissipation, resistive heaters, and electrodynamic dust shielding devices. Herein, we explore the microstructural evolution of electrically conductive, surface-localized nanocomposites (SLNCs) of chemically modified reduced graphene oxide and a set of thermoplastic polymers as a function of critical thermal properties of the substrate (melting temperature for semi-crystalline materials or glass transition temperature for amorphous materials). Selected offsets from critical substrate temperatures were used to produce SLNCs with conductivities between 0.6–3 S/cm and surface structures, which ranged from particle-rich, porous surfaces to polymer-rich, non-porous surfaces. We then demonstrate the physical durability of these electrically conductive SLNCs to expected stress conditions for flexible conductive materials in lunar applications including tension, flexion, and abrasion with lunar simulant. Small changes in resistance (*R*/*R*_0_ < 2) were measured under uniaxial tension up to 20% strain in high density polyethylene and up to 500 abrasion cycles in polysulfone, demonstrating the applicability of these materials as active and passive flexible conductors in exterior lunar applications. The tough, electrically conductive SLNCs developed here could greatly expand the use of polymeric materials in space applications, including lunar exploration, micro- and nano-satellites, and other orbital structures.

## 1 Introduction

In the lunar environment, contamination, electrostatic charging, and material wear due to interaction with lunar regolith is one of the major unsolved challenges for human habitation and exploration.^1−3^ Interaction with regolith can cause insulating materials to collect a static charge and electrostatic discharge (ESD) events can occur, potentially causing severe damage and safety risks in spacecraft,^4^ extra-terrestrial habitats, scientific equipment, and spacesuits.^5,6^ Integration of electrical conductivity into polymer substrates through electrically conductive fillers, small molecules, or intrinsically conductive polymers can provide protection from electrostatic charging by allowing surface charges to flow to an electronic ground.^7^ Conjugated carbon nanostructures—including carbon nanotubes (CNTs), graphene, graphene oxide (GO), and reduced graphene oxide (rGO)—are proving to be useful as the functional component in polymer nanocomposites for space applications due to their electrical conductivity, high stiffness, low atomic number (which reduces secondary radiation generation), and chemical stability in the space environment.^8−10^ These nanostructures are also more durable to the intense solar radiation, ultrahigh vacuum, wide temperature ranges, and high-speed impact events from micrometeoroids than small molecule additives and intrinsically conductive polymers.

Coating-based processing schemes, in which carbon materials are placed only at the surface of a composite structure, avoid some of the rheological and dispersion challenges associated with highly loaded blended nanocomposites, generally require less material, and achieve higher conductivity than their blended counterparts. Many strategies have been reported to form coatings or repeated multi-layers of carbon nanostructures on thermoplastic substrates including chemical vapor deposition,^11^ layer-by-layer (LBL) assembly,^12,13^ coextrusion,^14^ melt infiltration,^15^ and surface carbonization.^16^ Each of these methods produces coatings with varied microstructural characteristics—including thickness, surface roughness, chemical surface functionality, porosity, and particle alignment—based on the assembly technique. One space-tested method for making microns-thick, electrically conductive coatings is through surface carbonization in which a laser is used to locally carbonize the surface of a polymer substrate resulting in 50–200 μm thick graphitic foam structures with film resistivities ranging from 20 to 100,000 Ω/□.^16^ These structures can be converted to fully dense, composite structures through liquid-phase infiltration with a pre-polymer to create conductive materials with low sheet resistance values, down to 5 Ω/□, and smooth surface morphologies.^17,18^ These infiltrated composite structures have shown improved resistance to atomic oxygen (AO) degradation in low Earth orbit (LEO) providing ESD protection despite AO exposure due to their integrated carbon structures.

Surface-localized nanocomposites (SLNCs) represent a class of coatings in which regions near the surface, up to 10 s of μm, are composed of a nanocomposite structure, while regions away from the surface are primarily polymer. Liquid-phase infiltration of pre-formed particle layers, through wetting with pre-polymer,^17,19^ solvated polymer, or melt infiltration^20^ are common methods to form SLNCs.^21^ In one example, a randomly oriented CNT mat was compressed on the surface of a variety of heated thermoplastics, including high density polyethylene (HDPE) and polypropylene (PP), to infiltrate the CNT mat with molten polymer. Highly textured surfaces with sheet resistances of 10–1,000 Ω/□ were obtained.^15^ This approach has subsequently been employed in the production of superhydrophobic surfaces with improved wear behavior from similar partially infiltrated structures, but conductivity was not reported.^22^ Prior works on SLNCs of CNTs and other nanoparticles, do not provide a thorough evaluation of microstructural evolution relative to the thermal properties of the substrate materials or the electrical conductivity of these materials as a function of wear and deformation.^15,19,22^

In this work, we explore the microstructural evolution of electrically conductive, SLNCs of chemically modified reduced graphene oxide and a variety of thermoplastic polymers as a function of critical thermal properties of the substrate (melting temperature for semi-crystalline materials or glass transition temperature for amorphous materials). The substrates, HDPE, isotactic polypropylene (iPP), and polysulfone (PSU), used in this study were selected to cover a range of material properties to demonstrate that substrate viscosity can be used as a polymer agnostic control mechanism for SLNC structure development. Additionally, HDPE and PSU represent two space-relevant substrates taking on the roles of radiation protection and high-performance thermoplastic, respectively.^8^ We show that SLNCs formed on a variety of substrates contained similar conductive networks despite differences in polymer chemistry and microstructure, a feature that is difficult to achieve in melt blended systems. We then evaluate the physical stability of the conductive network of these SLNCs when subjected to flexion, tension, and abrasive wear to determine the suitability of these materials as flexible conductive surfaces for exterior lunar applications. Beyond static charge prevention and dissipation, the conductivities reported in this paper are sufficient for creating functional devices from plastic components, including dust mitigation systems,^23^ resistive heaters, electromagnetic interference shielding systems,^24^ and non-destructive evaluation systems.^16^

## 2 Materials and Methods

### 2.1 Synthesis of Dodecyl Modified Reduced Graphene Oxide (rGO-dd)

Reduced graphene oxide purchased from ACS Materials (GNCR0001; Pasadena, CA) was chemically modified with linear alkyl dodecyl substituents using the method described in our previous work.^25^ In brief, rGO was dispersed in *N*-methyl-2-pyrrolidone and reacted with 1-bromododecane (16970, Sigma-Aldrich) utilizing Williamson-ether synthesis conditions to create a chemically modified reduced graphene oxide (rGO-dd). After washing the product with methanol and drying, a powder material was obtained. The rGO precursor obtained from ACS Materials has a thickness of ∼1 nm, a monolayer diameter of 0.5–10 μm, a carbon content >82%, a conductivity >5 S/cm, and a surface area of 180 m^2^/g as measured by the Brunauer–Emmett–Teller (BET) method. Scanning electron microscopy (SEM) analysis of the functionalized and unfunctionalized particles showed an average single particle size of 2 μm measured across 40 particles. The minimum size measured was 0.9 μm, and the maximum individual particle size was 4 μm. A composite image of representative particle morphology and size for both functionalized and unfunctionalized particles is included in Figure S1. Fourier transform infrared (FTIR) spectra of the as-received rGO and the functionalized rGO-dd are included in Figure S2, and it shows the appearance of new features associated with alkyl chains appearing after functionalization.

### 2.2 Fabrication of Melt-Infiltrated Composites

**Figure 1:**
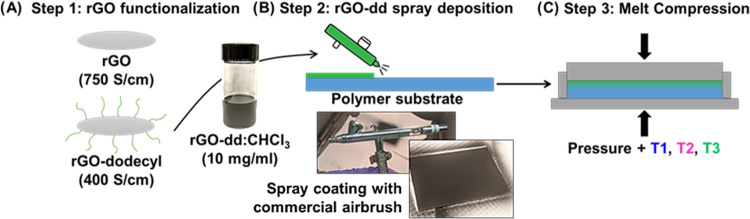
(A) Functionalization of rGO with dodecyl chains (rGO-dd) to improve dispersion and miscibility with polymer materials, dispersion of rGO-dd in CHCl_3_ for spraying. (B) Spray processing of a rGO-dd particle layer. (C) Melt infiltration is used to form a conductive SLNC without adhesives, solvents, pre-polymer, or pre-mixing.

rGO-dd was mixed with chloroform at a concentration of 10 mg/mL and sonicated for 30 min to produce a stable dispersion for spray casting, as shown in Figure 1A. Substrates were cleaned with an IPA rinse and allowed to dry before they were sprayed with the rGO-dd:CHCl_3_ solution using a gravity-fed airbrush (iwata Eclipse HP-CS; Portland, OR) to create a thin layer of rGO-dd with an areal density of approximately 0.01 mg/mm^2^. Airbrush and an as-sprayed film are shown in Figure 1B. The sprayed substrates were then placed in a mold between polytetrafluoroethylene (PTFE) films on the top and bottom faces as a mold release, shown schematically in Figure 1C. The assembled mold was heated to the selected processing temperature and put under 2.55 MPa of pressure using a heated press (Carver 4386; Wabash, IN). For each polymer, three processing temperatures were selected relative to the glass transition temperature (*T*_g_) or melting temperature (*T*_m_) depending on matrix morphology. The specific temperatures are given in Table S1 and as part of the Section 3. The press temperature was set slightly above the target temperature to achieve the correct mold temperature. The mold temperature was measured directly with a thermocouple adhered to the surface. The mold assembly was held at the selected temperature and pressure for 30 min to allow melt infiltration to occur before the platens and mold were cooled with recirculating water. For the HDPE, 1/16” (≈1.6 mm) thick sheets were obtained from McMaster-Carr. For PSU (*M*_w_ ∼ 35,000) and iPP (*M*_w_ ∼ 250,000), pellet materials were pressed into 1.5 mm thick sheets using the same mold configuration as the infiltration step. The resulting SLNC materials are designated as rGO-dd/HDPE, rGO-dd/iPP, and rGO-dd/PSU.

### 2.3 Differential Scanning Calorimetry

Measurements were made for each substrate to determine processing temperatures. A Discovery differential scanning calorimetry (DSC) (TA Instruments) was used to analyze a 5–8 mg sample of each polymer in the as-received state. Scans were collected at a heating rate of 10 °C/min over appropriate temperature ranges for each substrate, and data from the first heating cycle were used to select conditions.

### 2.4 Electrical and Electromechanical Characterization

Resistance measurements were made with a four-point line probe (Signatone SP4, 10 mm pin spacing) and a Keithley 2400 SourceMeter. Samples were measured in the as-produced state at five locations. Conductivity was calculated from these measured values using composite layer thickness measured from SEM cross sections, 40 μm for PSU and 20 μm for HDPE and iPP substrates. Average conductivity values and standard deviation are reported in Table 1. For resistance versus strain measurements, ASTM D638 Type V specimens were fabricated in which only the gage region was a SLNC structure and silver paste was applied at contact points at the edge of the gage region (spaced evenly for all samples). Alligator clips were applied to the silvered pads to make electrical contact, and a Keithley 2400 SourceMeter was used to monitor resistance in a two-point measurement mode. Samples were loaded in tension using an Instron 5566 load frame equipped with a 1 kN load cell and an extension rate of 1 mm/min. Strain was calculated from crosshead extension values.

**Table 1:** Average Conductivity Values and One Standard Deviation in Values for Each Substrate and Processing Condition^a^

	conductivity (S/cm)		
composite	LT	MT	HT
rGO-dd/HDPE	2.02 ± 1.2	2.98 ± 0.8	1.98 ± 1.4
rGO-dd/iPP	0.60 ± 0.09	0.85 ± 0.05	1.56 ± 0.06
rGO-dd/PSU	0.67 ± 0.09	0.79 ± 0.05	0.71 ± 0.2

a Conductivities were calculated assuming a film thickness of 20 μm for HDPE and iPP substrates and 40 μm for PSU substrates, as measured by SEM cross section.

### 2.5 Abrasion with Lunar Simulant

Abrasion testing was performed with a TABER rotary abrasion tester with a method adapted from Gaier et al. developed for abrasion testing of spacesuit fabrics.^26^ In short, S-39 abrasion wheels (soft leather wrapped wheels) and lunar highland simulant with a mean particle diameter of 2 μm (LHS-1, UCF Exolith Lab) were used to evaluate abrasion resistance of SLNC in the presence of lunar regolith.^27^ A 500 g counterweight was added to each abrasion arm to reduce head pressure. For each abrasion step, a separate sample was abraded (125, 250, 500, 1000 cycles) to reduce error due to sample re-insertion after measurement. Post abrasion, sheet resistances were measured by a four-point line probe (as above) at multiple locations perpendicular to the abrasion direction after gently brushing loose debris and regolith simulant from the surface. Conductivities were calculated as a function of original film thickness as determined by SEM cross section.

### 2.6 Scanning Electron Microscopy

A Phenom XL G2 SEM was used for imaging of SLNC structures and sprayed particle morphology. Top-down SEM views were produced using as-processed, uncoated samples. Cross-sectional samples of SLNCs were produced through freeze-fracturing. 50 mm × 5 mm strips were cut from melt-infiltrated coupons and submerged in liquid nitrogen for 20 min. Samples were then broken into two parts using tweezers to snap the sample, mounted to aluminum stubs, and sputter coated with gold to prevent charging. A Hitachi SU8010 SEM was used to collect images of individual particles for particle size analysis. Particles were dispersed under sonication at 10mg/L in water or CHCl_3_, for rGO and rGO-dd particles, respectively, and deposited on a lacey carbon-coated copper transmission electron microscopy (TEM) grid for imaging (Electron Microscopy Services, LC200-Cu).

### 2.7 Surface Profilometry

Surface profiles were obtained using a Bruker DektakXT stylus profilometer in mapping mode. A 1 mm × 1 mm region was scanned for each sample to show representative features. Mean roughness (*S*_a_) and root mean square roughness (RMS) values were calculated over the entire region using the calculation tools in Gwyddion.^28^

### 2.8 Raman Spectroscopy

A Renishaw inVia Raman microscope equipped with a 488 nm and a 785 nm laser was used to collect the Raman spectra, data from 488 nm scans are shown for iPP and HDPE, data from 785 nm scans are shown for PSU. Scans from the alternative laser configuration for each of these materials showed less distinct, lower intensity differentiating features and are excluded from this data set for clarity. Individual scan parameters were developed for each material to produce smooth spectra, and all composites and neat samples from a material were scanned with the same developed parameters.

### 2.9 Fourier Transform Infrared Spectroscopy (FTIR)

A Shimadzu IRAffinity spectrometer and an attenuated total reflectance (ATR) attachment were used to collect a scan range of 400 to 4000 cm^–1^, and an average of 20 scans was used for all samples. For Raman, FTIR-ATR, SEM, 4-pt resistivity, and profilometry, the melt-infiltrated substrates were used in their as-produced state.

## 3 Results and Discussion

Prior works on SLNCs of CNTs selected a single processing temperature for a variety of thermoplastics and provided limited explanation for observed differences in microstructural evolution.^15,19,22^ In this work, the Lucas–Washburn (L–W) model (eq 1) of melt infiltration was considered to understand the governing dynamics of the melt infiltration process with respect to substrate thermal properties.^29^ The L–W model was developed for the pressure-assisted capillary wetting of a solid, porous phase with a Newtonian liquid. In the area of nanocomposites, this model has traditionally been applied to the melt infiltration of ceramic and nanoceramic composites with Newtonian liquids, but it has shown to be applicable to nanocomposites systems based on carbon nanomaterials and non-Newtonian polymer melts.^15,29^ In this model, the penetration depth (*x*(*t*)) depends on the pore radius (*r*_p_), a particle–fluid surface energy interaction term (γ_lv_ cos(θ)), the viscosity of the infiltrate (η), and the duration of infiltration (*t*).^29^
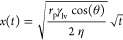
1

For this study, the infiltration time was held constant, and the pore radius was assumed to be consistent between samples. The surface energy term is expected to vary between the substrates selected for this study, but the range of this value will not exceed more than one order of magnitude given the limited range of values for γ_lv_ and cos(θ). In comparison, viscosity values will vary multiple orders of magnitude at temperatures just above critical substrate temperatures such as *T*_g_ and *T*_m_, for amorphous and semi-crystalline materials, respectively. This large dynamic range generates a variety of surface and near-surface microstructures ranging from particle-rich, porous structures to polymer-rich, non-porous structures.

**Figure 2:**
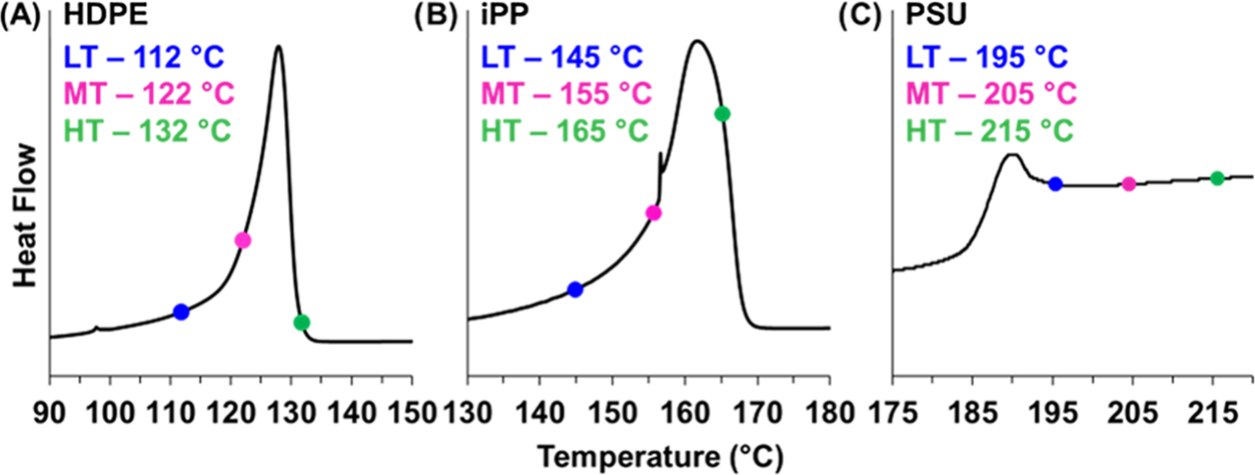
DSC plots showing the melting peak of (A) HDPE and (B) iPP, marked with the three processing temperatures used for each material. (C) DSC plot of the glass transition of PSU showing the three processing temperatures. Temperatures were selected to provide a wide range of viscosities to evaluate microstructural evolution as a function of substrate properties. Exothermic processes are indicated by negative heat flow values for all graphs.

For each substrate, three infiltration temperatures, at 10 °C increments, were selected to provide a range of viscosity values for a given matrix. For the semi-crystalline materials, HDPE and iPP, processing temperatures were selected to probe a range of temperatures in the melt regime. For the PSU matrix, three processing temperatures above the glass transition were selected. Processing temperatures for all materials are marked by the blue (low-temperature, LT), pink (middle-temperature, MT), and green (high-temperature, HT) dots on the thermographs in Figure 2; processing temperatures can also be found in Table S1.

For all lamination conditions, conductivities ranging from 0.6–3 S/cm were measured indicating that conductive composite surfaces with conductivities suitable for ESD and a variety of active and passive devices were created successfully in all processing conditions.^16^ The conductivity of all melt-infiltrated surfaces was analyzed by a 4-pt. in-line probe and are summarized in Table 1. Similar conductivities, within one order of magnitude, across all conditions and all materials may be attributed to the formation of a physical particle network during the spray deposition, which is not significantly altered during infiltration. If the percolated network structure was disrupted or interparticle spacing increased significantly during infiltration, the measured conductivities would drop many orders of magnitude as the matrix material is highly insulating. This result demonstrates that melt infiltration can be effectively used to create conductive particle networks in a variety of polymer systems without the use of in situ polymerization or solvent-assisted infiltration.

Despite the largely similar conductivity values, visual inspection of the SLNCs revealed an increase in surface gloss with increasing processing temperature indicating a change in the surface microstructure. Photographs of all composite surfaces are included in Figure S3. To evaluate the microstructural evolution of these SLNCs a multi-scale analysis approach was used. Profilometry was used to understand if increased surface gloss correlated to surface smoothing. RMS and *S*_a_ roughness values for all substrates can be found in Table S2. Profilometry plots used to calculate values and describe the evolution of surface roughness are provided in Figures S4 and S5. Generally, increasing processing temperature decreased surface roughness (RMS) from 1.28–1.69 μm in the low-temperature, matte SLNCs to approximately 0.8 μm in the high-temperature, glossy SLNCs. RMS values measured on HT HDPE substrates are excluded from this range due to the strong influence of the pressing configuration on the roughness values of this condition as detailed in Figure S5. Low surface roughness is a critical factor in enhancing the durability of flexible conductive coatings in the space environment. In lunar environments in particular, the presence of large amounts of highly abrasive regolith can result in rapid degradation of rough or porous surfaces where flaws and roughness can act as dust collection and degradation initiation points; a lesson learned during Apollo missions when EVA suits returned with unexpectedly high levels of abrasion and fiber fraying in the uncoated woven fabric materials. Prior studies on woven fabrics have shown that abrasion resistance is improved when exposed surfaces are smoothed and porosity is reduced.^30,31^ It is expected that the smooth coatings produced by the high-temperature processing conditions would retain ESD functionality longer than a comparable rough nanocomposite coating in the ablative and abrasive environments of low earth orbit (LEO) and the lunar surface, respectively.

**Figure 3:**
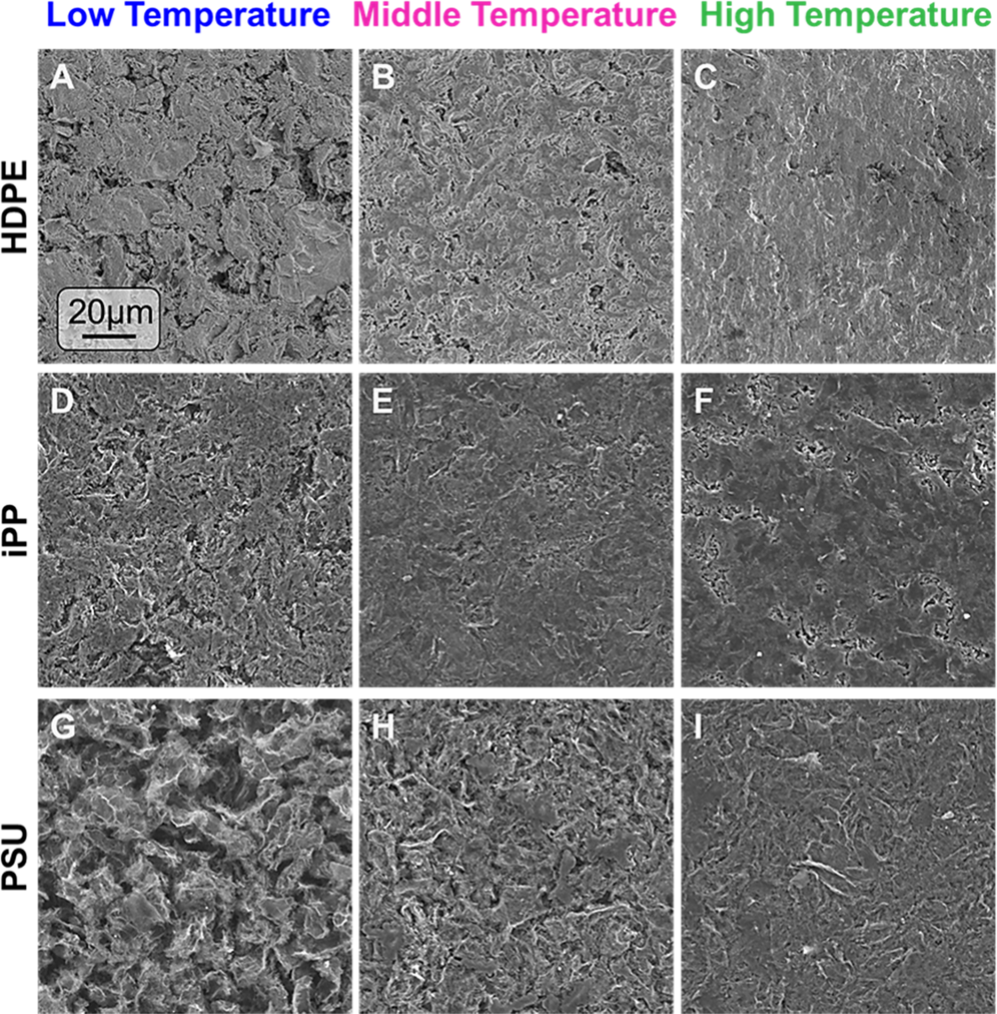
Top-view SEM images of the SLNCs processed at selected processing temperatures for (A–C) HDPE, (D–F) iPP, and (G–I) PSU substrates, highlighting the evolution of surface morphology with increased processing temperatures. In all materials, voids were more prominent at the LT condition and were significantly reduced at higher temperatures as polymer infiltration depth increases up to the thickness of the rGO-dd layer.

Top-down SEM images of SLNCs (Figure 3) were used to examine the presence of cracks or voids at the micron and sub-micron scale, which could entrap lunar regolith at the surface and to understand the mechanism for smoothing observed in the optical and profilometry analysis. For the HDPE and iPP SLNCs in the LT condition, shown in Figure 3A,D, respectively, large macrostructures on the order of 10–20 μm in diameter are visible. These stacked sheet structures appear similar to the rGO-dd nanoplatelets in the as-sprayed morphology (Figure S6); however, the LT treatment did result in the large void spaces observed in as-sprayed coating being filled with polymer (Figure S6). This particle-rich, polymer-poor morphology explains the high surface roughness measured by profilometry and their matte appearance. In contrast, the LT condition in PSU, shown in Figure 3G, showed no visual evidence of void filling at the surface by the infiltrating matrix, possibly due to the higher-expected infiltrate viscosity of a matrix near its *T*_g_ as compared to matrices near their melting regime.

Images in Figure 3B,E,H are taken at the MT condition. All three materials showed improved surface consolidation and reduced void content that yielded a more uniform surface appearance, in agreement with the surface smoothing and gloss increase observed previously. For the HDPE and iPP substrates in Figure 3B,E, fewer distinct rGO-dd macrostructures were observed, and the remaining void edges were more rounded in appearance, suggesting the polymer had infiltrated all large pores and begun filling smaller voids. For PSU, the MT condition (Figure 3H) retained a slightly wrinkled surface texture and a greater number of voids, suggesting that the extent of infiltration progress was smaller than the iPP and HDPE substrates at the MT condition due to high expected infiltrate viscosity.

For the SLNCs processed at the HT condition, the HDPE and PSU surfaces in Figure 3C,I exhibited no micron scale voids and large regions of smooth, polymer-rich surface. These images agree with the glossy appearances and reduced surface roughness obtained at this processing temperature. While some voids were still present on the iPP surface in Figure 3F, they were fewer in number and smaller in size than the LT and MT conditions, which indicated an increased extent of infiltration.

While no voids were present in the PSU HT condition (Figure 3I), there were still distinct wrinkled features covering most of the surface. This texture resembles that of the rGO-dd sheets (Figure S1) partially protruding from the surface and suggests that the composite formation process of the PSU may progress differently than the iPP and HDPE substrates where sheet edges are less apparent after infiltration. One factor may be the higher-expected viscosity of PSU near its *T*_g_ compared to the iPP and HDPE viscosities near their *T*_m_. The surface energy mismatch of the polymer and particle or the amorphous polymer matrix structure may also play a role in these morphological differences. Note that there were a few small, distributed regions in Figure 3I that appeared to have a smoother surface texture similar to that of the HT condition iPP and HDPE substrates after HT processing. These regions are presumed to have greater polymer content and will be discussed further in the Raman spectroscopy data discussed below. The nearly complete infiltration of all three materials in the HT condition provides an appropriate surface microstructure to reduce the impact of abrasive regolith exposure in lunar environments and ablative AO exposure in LEO environments.

**Figure 4:**
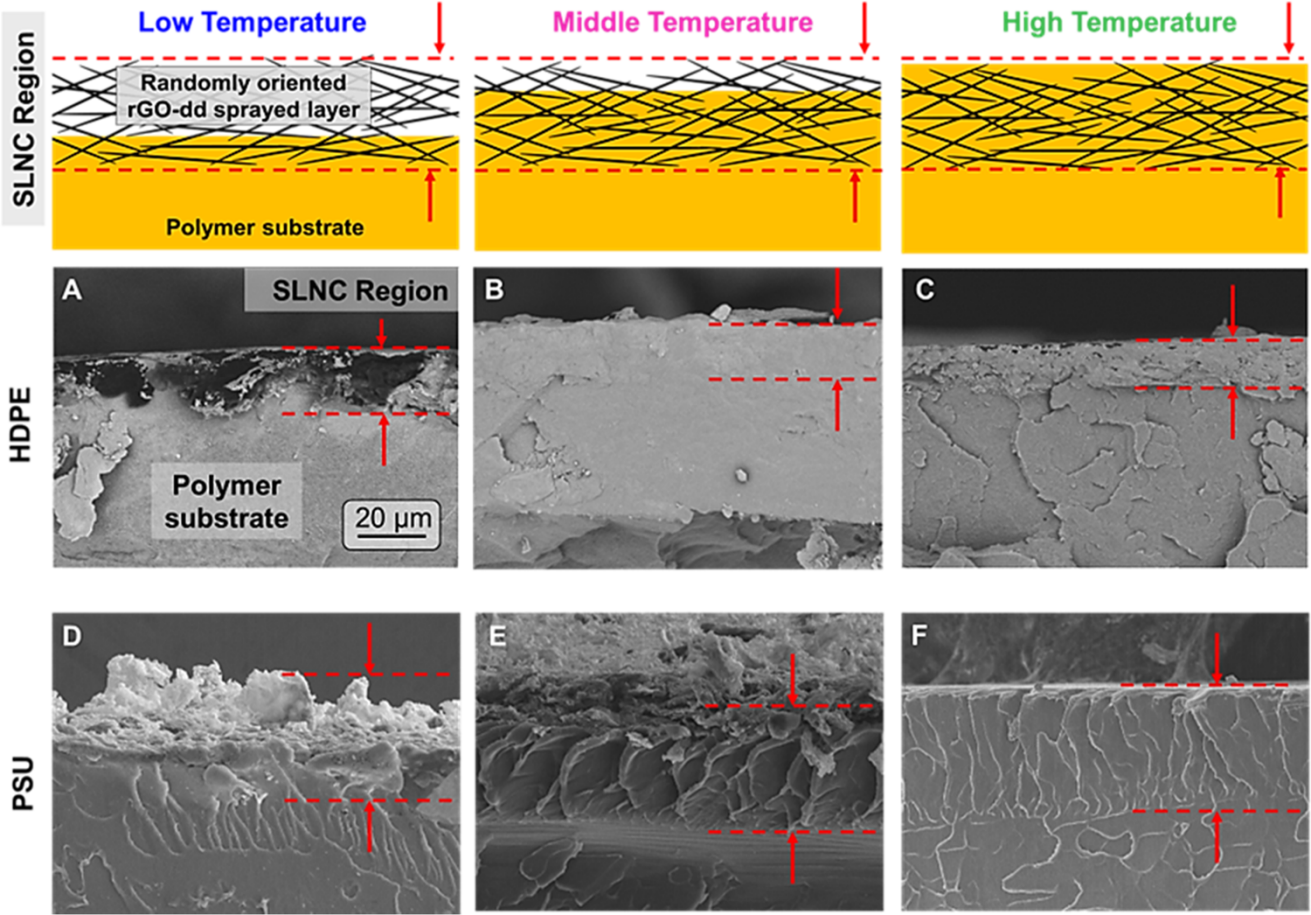
(Top) Schematic images of cross-sectional microstructure where black needle-shaped objects are rGO-dd platelets and yellow solid regions are polymer substrates. Red dashed regions in images and schematic highlight consistent rGO-dd network thicknesses at all processing stages. (A–C) Cross-sectional SEM images of the composite layer formation for HDPE and (D–F) PSU substrates show the progression of infiltration as a function of temperature for both materials. Low-temperature processing results in shallow infiltration depths and particle-rich surfaces. Increased temperatures resulted in reduced matrix viscosity and increased infiltration depth. Fracture edges were coated in gold to allow for imaging of the non-conductive polymer regions below the coating.

Cross-sectional SEM images of the HDPE and PSU substrates were collected to further characterize the infiltration dynamics and microstructure evolution. iPP was excluded from this portion of the study as all other measures indicated highly similar behavior as HDPE. In the LT condition for both substrates (Figure 4A,D), free-standing rGO-dd particles and large internal voids indicating low infiltration depths were observed. This result agrees with the high surface roughness, matte appearance, and porous surface observed in top-view SEM images. As the processing temperature was increased to the MT condition and substrate viscosity decreased, both substrates had more complete infiltration (Figure 4B,E) as expected from evaluation of the L–W model (eq 1). In the case of HDPE, there were no visible rGO-dd macrostructures or voids along the entire 5 mm length of the fractured edge and a few particle edges protruding from the surface, indicating nearly complete infiltration of surface and sub-surface void spaces. This observation agrees with the reduced void content, surface smoothing, and increased gloss observed previously. For the MT PSU substrate, the cross-sectional SEM analysis provided further evidence of near-surface voids and partially embedded rGO-dd particles due to the limited infiltration depth of the polymer substrate attributed to the higher-expected viscosity of PSU around *T*_g_ versus HDPE around *T*_m_.

At the HT condition for PSU and HDPE (Figure 4C,F), there is no longer evidence of individual rGO-dd nanoparticles within the SLNC layer or partially embedded particles protruding from the surface, indicating complete infiltration of the porous particle network. In the MT and HT condition images, the SLNC region can be identified by a difference in fracture surface texture (marked by dashed lines) attributed to the expected difference in mechanical properties between the SLNC region and the neat polymer substrate. Additionally, these regions are approximately the same thickness between conditions and are comparable to the largely un-infiltrated particle layer observed in the low processing condition, approximately 10–15 μm for the HDPE and 40–50 μm for the PSU substrates. This consistency provides further support that the conductive network formed during rGO-dd spraying remains largely undisturbed during the infiltration process.

The low-porosity, smooth, solid surfaces produced by the HT condition should limit the infiltration paths of lunar dust into exterior polymer surfaces enhancing durability. The homogeneous distribution of conducting particles throughout the 10–40 μm thickness should also be beneficial for maintaining ESD capability in the event of surface damage, abrasion, or ablation. This is a significant advantage when compared to thin, metallic atomic layer deposited (ALD) coatings typically employed to enhance surface discharge and provide atomic oxygen protection in space environments. ALD coatings can be easily damaged during transport, assembly, and use, which will disrupt the conductive pathway and potentially result in unwanted electrical charging or degradation.^32^ Additionally, by linking infiltration dynamics to critical thermal transitions in thermoplastics, this work demonstrates how simple material characterization (e.g., DSC) can be used to rapidly select effective processing conditions for SLNCs on a variety of thermoplastics to produce predictable microstructures and form robust conductive networks.

To measure the local chemical character of the SLNCs and further describe the surface structure observed through SEM and profilometry, two surface-sensitive spectroscopy techniques, ATR-FTIR and Raman, were used. While both techniques probe surface and sub-surface material, the thick nature of the coatings ensures that the resultant signals are descriptive of the near-surface composite composition and not the underlying substrate. When partially infiltrated films are examined using these techniques, we expected that only signals associated with the rGO-dd particles would be present in the micron range at the surface. As infiltration increased to the thickness of the sprayed particle layer, we expected to observe signals from both the infiltrate and the particles as both components should be present at the surface of the nanocomposite structure.

**Figure 5:**
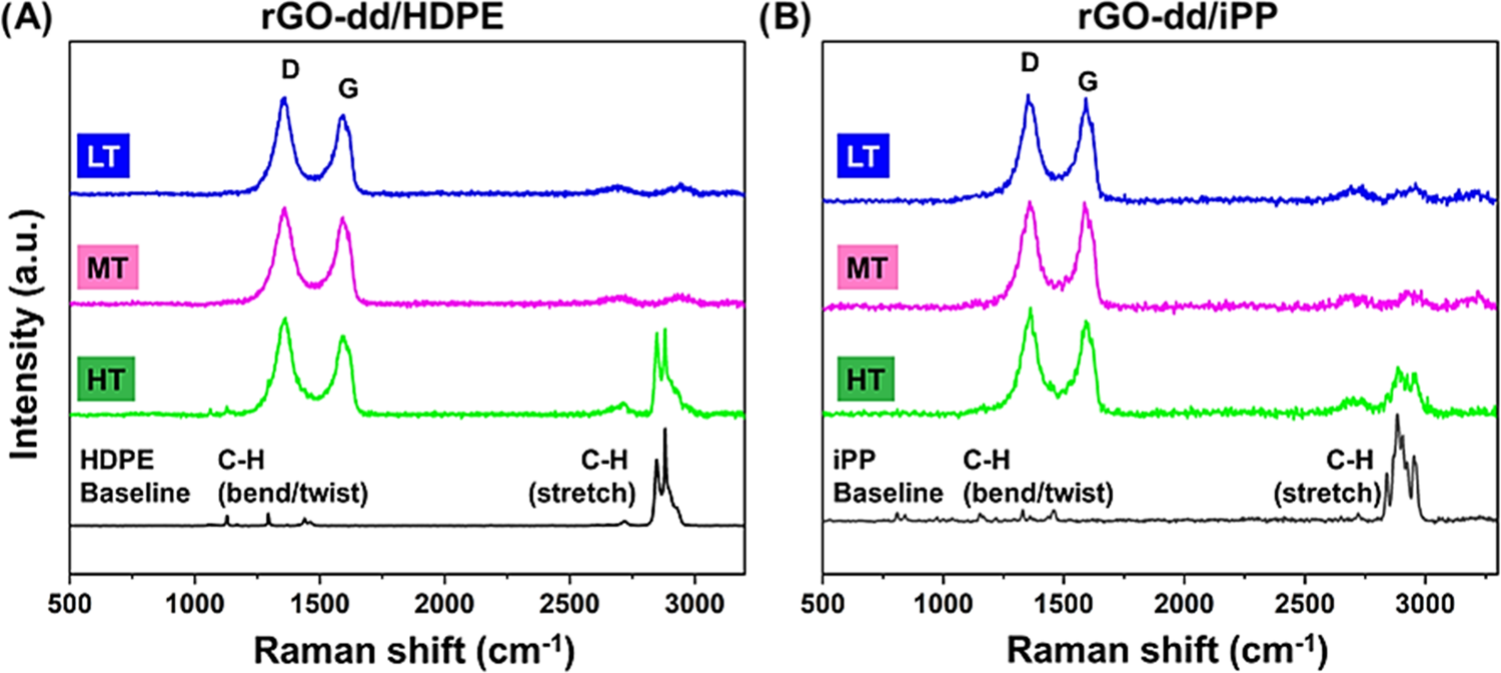
Raman spectra of composite surface for each processing condition on (A) HDPE and (B) iPP. Signals related to the polymer (2700–2900 cm^–1^) only appeared in the high-temperature conditions when the infiltration depth was equal to the sprayed layer thickness.

Raman spectra of HDPE and iPP SLNCs (Figure 5) confirm the expected trend as the composites transition from partial to complete infiltration. While many peaks of rGO-dd, HDPE, and iPP are overlapping, the rGO-dd is marked by strong peaks at 1300 and 1700 cm^–1^. iPP and HDPE present weak peaks between 1085–1421 cm^–1^ related to C–H bending vibrations^33^ and stronger peaks in the 2700–2900 cm^–1^ region related to C–H stretching, as shown in the baseline HDPE and iPP plots in Figure 5. The LT and MT conditions for these substrates showed strong peaks associated with rGO-dd and very little signal from the polymer, indicating a particle-rich surface chemistry. Only at the HT condition are strong polymer peaks observed in agreement with the optical images, profilometry, and SEM results presented above. Notably, all spectra show a strong rGO-dd signal, indicating that, even at full infiltration depth, a significant fraction of the material is rGO-dd particles, which agrees with the similar conductivity measured on matte LT and glossy HT surfaces. Surfaces rich in conjugated carbon materials have shown improved durability when exposed to AO and other space hazards due to the chemical stability of the conjugated structure as compared to common polymer matrix materials.^18^

Some variability in the intensity of the peaks at 2700–2900 cm^–1^ was observed across the surface, but all locations showed a distinct polymer signal and uniform optical appearance. The same trend was observed in ATR-FTIR analysis of HDPE, and the data are presented in Figure S7. While the characteristic FTIR peaks are not as distinct as the Raman spectra, the characteristic signal of the polymer substrate appears at higher processing temperatures while rGO-dd signals are present across the range of melt infiltration temperatures.

**Figure 6:**
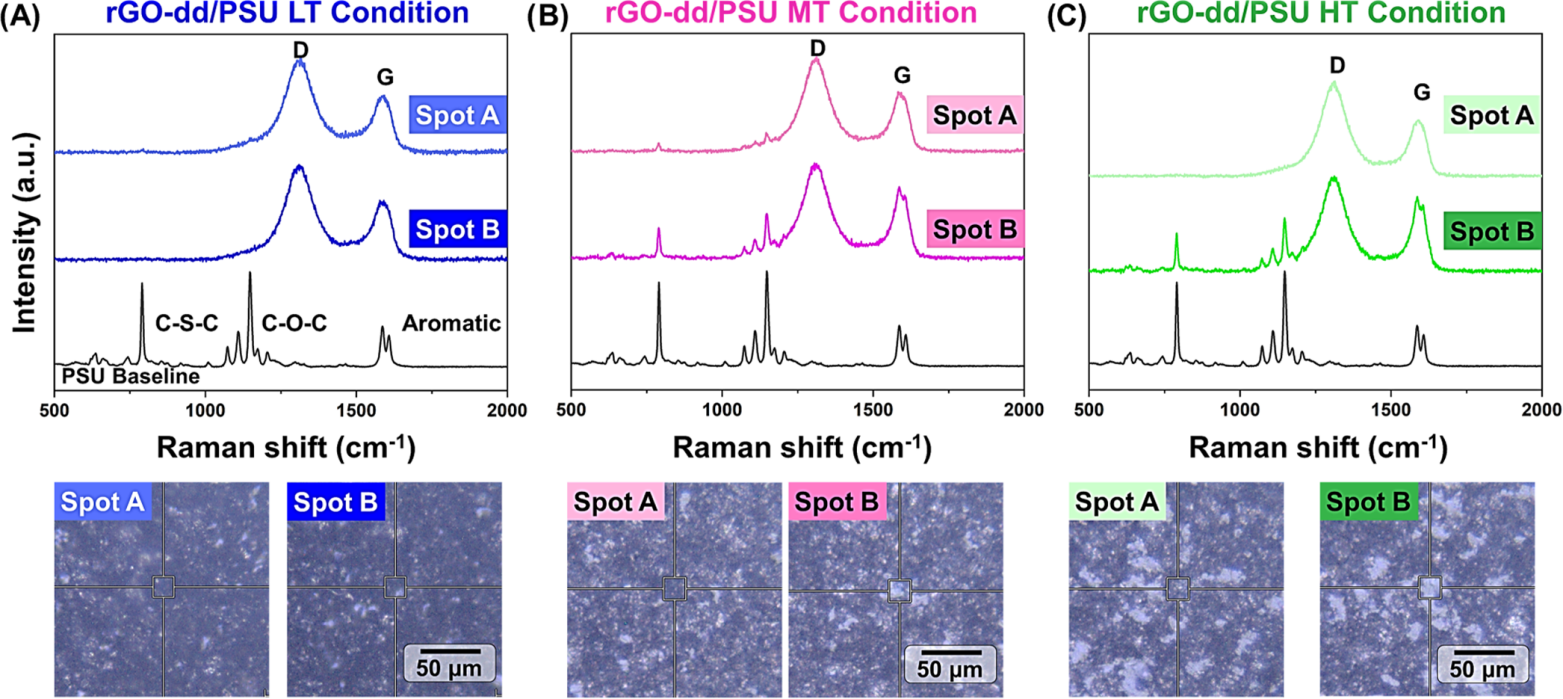
Raman spectra of composite surface for PSU at (A) LT, (B) MT, (C) and HT conditions with corresponding optical images of scanned regions. A distinct speckled appearance that evolves with processing temperature is seen in the optical images and corresponds to regions with more (silvery regions) or less polymer character. Notably, all regions still show a significant rGO-dd signal (1300, 1700 cm^–1^).

For rGO-dd/PSU surfaces, significant chemical and optical heterogeneity is observed at the MT and HT processing conditions, which was not observed for any iPP and HDPE surfaces. Figure 6 shows Raman spectra at two locations for each processing condition with corresponding optical images of the scanned regions. For the LT condition, though a slight speckle pattern was observed in the optical images, all spots show only the rGO-dd character. At the MT condition, the speckle diameter appeared to grow while the density and inter-regions spacing remained similar to the LT condition. These larger speckle regions exhibited more PSU character but retained a strong rGO-dd signal.^34^ In the HT condition, speckle regions appear to have partially fused into a smaller number of large regions. Similar to the MT condition, these regions show both PSU and rGO-dd character. Further investigation is needed to understand if this change in mechanism from uniform infiltration to spatially varied infiltration is primarily driven by differences in the particle–matrix interaction energy for aromatic polymers and polyolefins, the difference in viscosity between amorphous polymers at *T*_g_ and semi-crystalline polymers at *T*_m_, or other unexplored factors.

To understand how infiltrated layers were affected by mechanical stress, bending and tensile testing were performed on the MT and HT rGO-dd/HDPE substrates while measuring the two-point resistance between silver paste contact points. The rGO-dd/HDPE polymer system was selected for analysis as it is far above its *T*_g_ at room temperature and can undergo large plastic deformations before fracture. While maintaining electrical conductivity under mechanical stress is important for the durability of soft, electrically conductive materials in passive applications such as ESD,^13^ measuring the exact response to strain is critical for active device applications such as electrodynamic dust shielding, meteoroid impact detection, and strain sensing for which changes in resistance can result in altered device performance or anomalous readings.^35^

**Figure 7:**
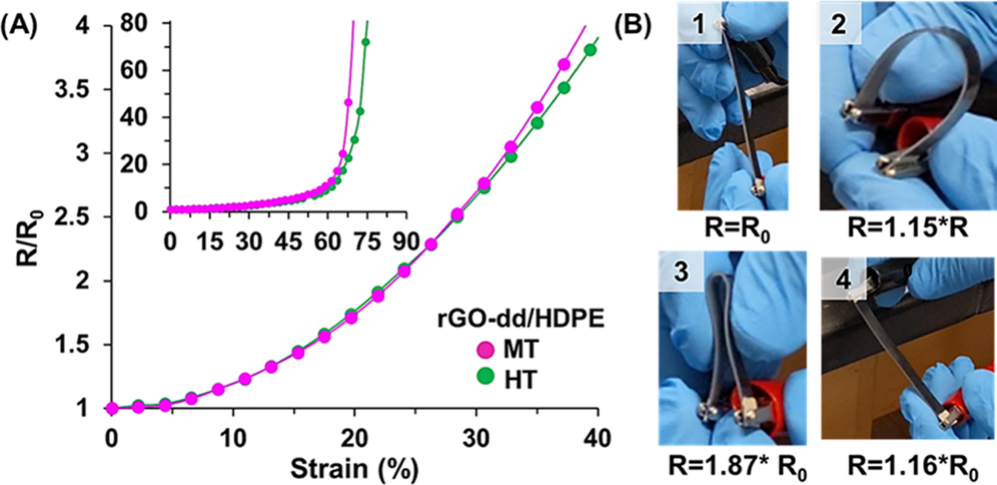
(A) Normalized two probe resistance values measured during uniaxial tension for MT (magenta) and HT (green) processing conditions in the region around the ultimate tensile strength. (inset) Normalized two probe resistance values measured for a wider range of strain values up to substrate failure. (B) Four stages of manual bending showing increased resistance during bending up to the substrate’s folding limit followed by recovery of conductivity upon unbending.

An analysis of the electromechanical testing in Figure 7A indicates that the rGO-dd/HDPE composites produced at the MT and HT condition were robust in tension with less than an order of magnitude increase in resistance (*R*/*R*_0_ < 2) at the point of ultimate tensile strength (UTS), which occurs around 20% strain. At this value, samples would still have sufficient conductivity to perform as ESD surfaces and in some active device configurations. Despite large deformation of the tensile samples beyond the UTS, a rapid rise in resistance (to the measurement limit of 10^9^ Ω) does not occur until 55–60% strain for both infiltration conditions, indicating the percolated network in the SLNC structure is tolerant to large deformations in the underlying substrate in comparison to metallic thin films, which often experience rupture at <10% strain. Specimen fracture typically occurs at 85–90% strain. The high extensibility of these conductive networks would allow SLNCs to be used in scenarios where repeated flexion or extension is required such as inflatable habitats and structures, spacesuits, and flexible robotic components, where traditional ALD films may not be reliable.

In Figure 7B, the rGO-dd/HDPE sample produced at the HT condition was manually folded and unfolded. Normalized resistance values were recorded at intermediate bending conditions. After folding, a permanent 16% increase in resistance is measured, but the sample remained conductive and no physical damage (e.g., cracking or crazing) was observed on the surface. The results of these electromechanical tests provide further evidence that the HDPE materials have formed a fully infiltrated composite structure at these processing conditions, allowing electrical contact to be maintained between the nanoparticles during extension and flexion.

**Figure 8:**
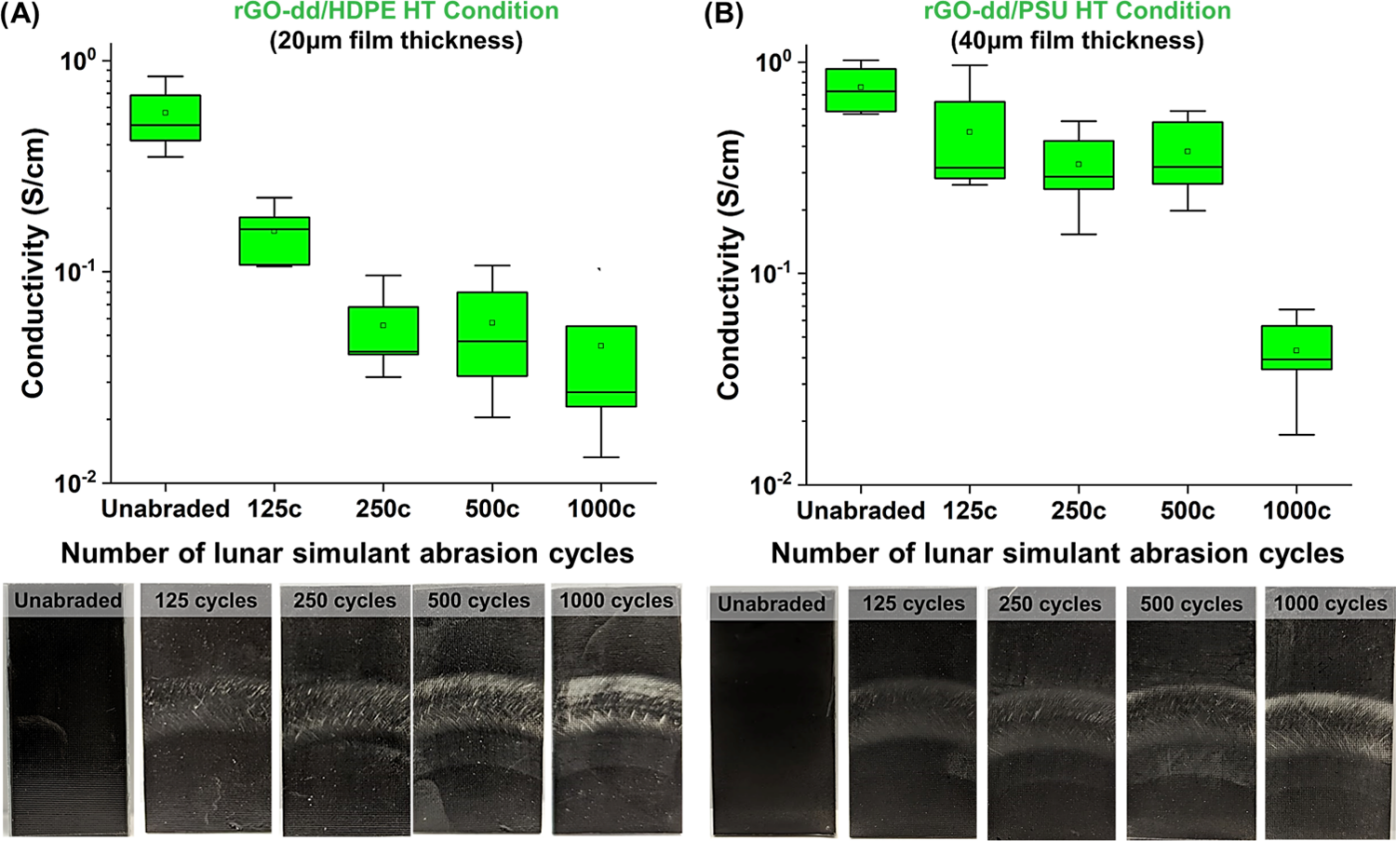
Conductivity versus the number of abrasion cycles in the presence of lunar simulant for (A) HT rGO-dd/HDPE and (B) HT rGO-dd/PSU substrates. (lower) Images of the substrates used to measure conductivity after abrasion. The conductivity of both substrates decreased with abrasive wear and corresponded with growing regions of the exposed polymer substrate.

In the lunar environment, exterior materials will be in constant contact with lunar regolith due to the rarified atmosphere and low gravity, which promotes lofting and movement of the dust during human and solar activity. Consideration of lunar regolith abrasion on material performance properties is critical to understanding the long-term durability of active and passive devices. Gaier et al. previously developed a modified Taber abrasion test for assessing the durability of spacesuit fabrics by matching degradation behavior on ground-tested fabrics with actual exposed fabric samples returned during the Apollo missions.^26^ A similar method was used here to evaluate degradation in conductivity as a function of lunar regolith abrasion (Figure 8).

HT rGO-dd/HDPE and HT rGO-dd/PSU were tested for this portion of the study as both substrates showed full infiltration during prior analysis and are polymers frequently used in space applications. The conductivity of the HT HDPE substrate decreased after only 125 abrasion cycles; however, it appears to stabilize at ≈5 × 10^–2^ S/cm with additional cycles (Figure 8A). The HT PSU samples demonstrated more robust conductivity with less visual wear up to 500 cycles, but a decline in conductivity was observed at 1,000 cycles to ≈4 × 10^–2^ S/cm (Figure 8B). In both materials, significant visual changes occur after 1000 cycles, which are attributed to mechanical deformation from abrasion. Nevertheless, even the most abraded regions (based on appearance) retain surface conductivities that are many orders of magnitude higher than the substrate conductivity (<10^–17^ S/cm), indicating that despite abrasive wear a conductive network is still present at the surface. Additionally, the conductivity values shown in Figure 8 are calculated from measured 4-pt. line probe values using a constant film thickness of 20 μm for HDPE and 40 μm for PSU. It is expected that abrasive wear will thin the coatings as particles and polymer are removed, resulting in a thinner conductive coating and higher resistance despite similar resistivity of the remaining conductive network.

To offer more context regarding the implications of these studies, it is germane to note that the abrasion pressures used in this study are significantly higher than expected for many lunar applications, particularly where hard body contact is not expected to occur often, for example, on many surfaces of habitat structures. To assess non-abrasive contact, a simulant was deposited and then removed by tapping the substrate against a hard surface. Much of the dust was easily removed by these processes, and no notable change in conductivity was observed despite some residual dust on the surface.

## 4 Conclusions

In this study, we have developed a simple fabrication method for mechanically robust, surface-localized, conductive coatings of rGO-dd nanocomposites on various polymer substrates through melt infiltration. SEM imaging, profilometry, and Raman spectroscopy were used to assess the degree of infiltration as a function of processing temperature relative to characteristic material temperatures (e.g., *T*_m_, *T*_g_). SLNC structures ranged from particle-rich, porous surfaces to polymer-rich, non-porous surfaces while maintaining similar conductivity due to the pre-established conductive particle network. Cross-sectional images show that fully infiltrated composites with no visible particle aggregates are reliably produced in the high-temperature processing condition regardless of the substrate material. This study provides a path to the rapid development of processing conditions based on simple material characterization (DSC) for highly loaded conductive nanocomposite surfaces without detailed consideration of matrix–particle compatibility. Chemical characterization of the surfaces at various processing temperatures was consistent with the proposed infiltration model in which higher processing temperatures, and resultant lower viscosities, increased the infiltration depth of the polymer substrate into the sprayed nanoparticle film. It is expected that processing times could be significantly shortened by increasing the processing temperatures beyond those explored herein.

The durability of these materials as flexible conductors for space applications was evaluated by electromechanical testing in tension and flexion during which these SLNCs structures maintain conductivity up to the point of plastic deformation (*R*/*R*_0_ < 2 at 20% strain on HDPE). Modified Taber abrasion studies using lunar simulant demonstrate that sufficient electrical conductivity for ESD protection can be maintained up to 1000 abrasion cycles on PSU (*R*/*R*_0_ ∼ 20, 0.4 × 10^–2^ S/cm). In summary, these results demonstrate a material agnostic method for microstructural control of SLNCs and the generation of tough, thick, electrically conductive SLNCs suitable for ESD in space environments and potential integration into active devices for space applications. Patterned versions of these materials have also been used to create fully integrated electrodynamic dust shielding an active device that can robustly remove lunar regolith in high vacuum environments. The results of this associated work have been reported elsewhere.^36^

Future work may include a comparison of these physical durability properties with other typical ESD protection coatings, including small molecule additives and metallic thin films. This would include an evaluation of the high and low-temperature electrical conductivity at temperatures relevant to the space environment and an in-depth analysis of mechanical properties and failure modes of these highly loaded SLNC structures compared to the substrate materials. On-going efforts to produce rGO with alternative pendant functionalities and additional infiltration trials will provide a closer examination of how particle–matrix surface energy differences influence the heterogeneity of the SLNC structure. These studies will also expand the palette of materials to include thermoplastics with alternative physical and chemical structures, such as partially fluorinated polymers, that are of specific interest to the space community.

## Supporting Information Available

Detailed processing temperatures for each material and treatment condition; SEM images of representative rGO-dd particles used for particle size analysis; FTIR-ATR spectra of rGO and rGO-dd particles; photographs of nanocomposite surfaces for all substrates and treatment conditions; tabulated roughness data for all conditions and corresponding surface roughness plots; additional profilometry data for HDPE substrates; SEM image of the as-sprayed particle morphology; FTIR-ATR spectra not included in the main text (PDF)

## References

[ref1] Weiss P.; Mohamed M. P.; Gobert T.; Chouard Y.; Singh N.; Chalal T.; Schmied S.; Schweins M.; Stegmaier T.; Gresser G. T.; Groemer G.; Sejkora N.; Shumit D.; Rampini R.; Hołyńska M. Advanced materials for future lunar extravehicular activity space suit. Adv. Mater. Technol. 2020, 2000028 10.1002/admt.202000028.

[ref2] Jackson T. L.; Farrell W. M.; Zimmerman M. I. Rover wheel charging on the lunar surface. Adv. Space Res. 2015, 55, 1710–1720. 10.1016/j.asr.2014.12.027.

[ref3] Calle C. I.; Mazumder M. K.; Immer C. D.; Buhler C. R.; Clements S.; Lundeen P.; Chen A.; Mantovani J. G. In Electrodynamic dust sheild for surface exploration activities on the Moon and Mars; In 57th International Astronautical Congress, 2006.

[ref4] Grard R.; Knott K. Spacecraft charging effects. Space Sci. Rev. 1983, 34, 289–304. 10.1007/BF00175284.

[ref5] Farrell W. M.; Jackson T. L.; Marshall J. R.; Delory G. T. In The Need for Conductive Space Suits: a Summary of DREAM 2 Findings; NESF, 2015.

[ref6] Farrell W. M.; McLain J. L.; Zimmerman M. I.; Hartzell C. M.; Fester Z. T. In Lunar Dust-to-Suit Electrostatic Interactions: Insulating vs. Conducting Space Suits; Lunar Dust Workshop, 2020.

[ref7] de Souza Vieira L.; dos Anjos E. G. R.; Verginio G. E. A.; Oyama I. C.; Braga N. F.; da Silva T. F.; Montagna L. S.; Rezende M. C.; Passador F. R. Carbon-based materials as antistatic agents for the production of antistatic packaging: a review. J. Mater. Sci.: Mater. Electron. 2021, 32, 3929–3947. 10.1007/s10854-020-05178-6.

[ref8] Nambiar S.; Yeow J. T. Polymer-composite materials for radiation protection. ACS Appl. Mater. Interfaces 2012, 4, 5717–5726. 10.1021/am300783d. 23009182

[ref9] Kleiman J.; Iskanderova Z.; Krishtein L.; Dennison J. R.; Wood B.; Grigorievsky A.; Best C. Ion beam-treated space polymers: long-term stability in GEO-simulated environments. CEAS Space J. 2021, 13, 433–443. 10.1007/s12567-021-00361-9.

[ref10] Marsden A. J.; Papageorgiou D. G.; Vallés C.; Liscio A.; Palermo V.; Bissett M. A.; Young R. J.; Kinloch I. A. Electrical percolation in graphene–polymer composites. 2D Mater. 2018, 5, 032003 10.1088/2053-1583/aac055.

[ref11] Vlassiouk I.; Polizos G.; Cooper R.; Ivanov I.; Keum J. K.; Paulauskas F.; Datskos P.; Smirnov S. Strong and Electrically Conductive Graphene-Based Composite Fibers and Laminates. ACS Appl. Mater. Interfaces 2015, 7, 10702–10709. 10.1021/acsami.5b01367. 25919004

[ref12] Lee T.; Min S. H.; Gu M.; Jung Y. K.; Lee W.; Lee J. U.; Seong D. G.; Kim B.-S. Layer-by-layer assembly for graphene-based multilayer nanocomposites: synthesis and applications. Chem. Mater. 2015, 27, 3785–3796. 10.1021/acs.chemmater.5b00491.

[ref13] Shim B. S.; Zhu J.; Jan E.; Critchley K.; Kotov N. A. Transparent conductors from layer-by-layer assembled SWNT films: importance of mechanical properties and a new figure of merit. ACS Nano 2010, 4, 3725–3734. 10.1021/nn100026n. 20552974

[ref14] Mun S. C.; Kim S. I.; Kim M. J.; Macosko C. W. Imprinting graphene on polymer substrates via coextrusion. Ind. Eng. Chem. Res. 2020, 59, 15929–15935. 10.1021/acs.iecr.0c01978.

[ref15] Peng M.; Liao Z.; Qi J.; Zhou Z. Nonaligned carbon nanotubes partially embedded in polymer matrixes: a novel route to superhydrophobic conductive surfaces. Langmuir 2010, 26, 13572–13578. 10.1021/la101827c. 20695606

[ref16] Li J. T.; Stanford M. G.; Chen W.; Presutti S. E.; Tour J. M. Laminated laser-induced graphene composites. ACS Nano 2020, 14, 7911–7919. 10.1021/acsnano.0c02835. 32441916

[ref17] Loeblein M.; Bolker A.; Tsang S. H.; Atar N.; Uzan-Saguy C.; Verker R.; Gouzman I.; Grossman E.; Teo E. H. 3D graphene-infused polyimide with enhanced electrothermal performance for long-term flexible space applications. Small 2015, 11, 6425–6434. 10.1002/smll.201502670. 26479496

[ref18] Shivakumar R.; Bolker A.; Tsang S. H.; Atar N.; Verker R.; Gouzman I.; Hala M.; Moshe N.; Jones A.; Grossman E.; Minton T. K.; Teo E. H. T. POSS enhanced 3D graphene - polyimide film for atomic oxygen endurance in low Earth orbit space environment. Polymer 2020, 191, 122270 10.1016/j.polymer.2020.122270.

[ref19] Wang S.; Haldane D.; Liang R.; Smithyman J.; Zhang C.; Wang B. Nanoscale infiltration behaviour and throughthickness permeability of carbon nanotube buckypapers. Nanotechnology 2013, 24, 015704 10.1088/0957-4484/24/1/015704. 23221271

[ref20] Huang Y.-R.; Jiang Y.; Hor J. L.; Gupta R.; Zhang L.; Stebe K. J.; Feng G.; Turnerb K. T.; Lee D. Polymer nanocomposite films with extremely high nanoparticle loadings via capillary rise infiltration (CaRI. Nanoscale 2015, 7, 798–805. 10.1039/c4nr05464d. 25436973

[ref21] Harito C.; Bavykin D. V.; Yuliarto B.; Dipojono H. K.; Walsh F. C. Polymer nanocomposites having a high filler content: synthesis, structures, properties, and applications. Nanoscale 2019, 11, 4653–4682. 10.1039/c9nr00117d. 30840003

[ref22] Hejazi I.; Sadeghi G. M. M.; Jafari S. H.; Khonakdar H. A.; Seyfi J.; Holzschuh M.; Simon F. Transforming an intrinsically hydrophilic polymer to a robust self-cleaning superhydrophobic coating via carbon nanotube surface embedding. Mater. Des. 2015, 86, 338–346. 10.1016/j.matdes.2015.07.092.

[ref23] Manyapu K. K.; Peltz L.; De Leon P. Extending the utilization of dust protection systems using carbon nanotube embedded materials for lunar habitats for exploration missions. J. Space Saf. Eng. 2019, 6, 248–255. 10.1016/j.jsse.2019.10.001.

[ref24] Abbasi H.; Antunes M.; Velasco J. I. Recent advances in carbon-based polymer nanocomposites for electromagnetic interference shielding. Prog. Mater. Sci. 2019, 103, 319–373. 10.1016/j.pmatsci.2019.02.003.

[ref25] Seibers Z. D.; Brim E.; Pittelli S. L.; Beltran E.; Shofner M. L.; Reynolds J. R. Readily dispersible chemically functionalized reduced graphene oxide nanosheets for solution-processable electrodes and conductive coatings. ACS Appl. Nano Mater. 2020, 3, 11455–11464. 10.1021/acsanm.0c02539.

[ref26] Gaier J. A.; Meador M. A.; Rogers K. J.; Sheehy B. H. In Abrasion of Candidate Spacesuit Fabrics by Simulated Lunar Dust; NASA/TM-2009-215800, 2009.

[ref27] Clendenen A. R.; Aleksandrov A.; Jones B. M.; Loutzenhiser P. G.; Britt D. T.; Orlando T. M. Temperature programmed desorption comparison of lunar regolith to lunar regolith simulants LMS-1 and LHS-1. Earth Planet. Sci. Lett. 2022, 592, 117632 10.1016/j.epsl.2022.117632.

[ref28] Klapetek P.; Necas D.; Anderson C. In Data processing and analysis; Gywddion User Guide, 2022.

[ref29] de Jongh P. E.; Eggenhuisen T. M. Melt infiltration: an emerging technique for the preparation of novel functional nanostructured materials. Adv. Mater. 2013, 25, 6672–6690. 10.1002/adma.201301912. 24014262

[ref30] Christoffersen R.; Lindsay J. F.; Noble S. K.; Meador M. A.; Kosmo J. J.; Lawrence J. A.; Brostoff L.; Young A.; McCue T. In Lunar Dust Effects on Spacesuit Systems: Insights from the Apollo Spacesuits, NASA/TP-2009-214786; NASA, 2009.

[ref31] Gaier J. R.; Creel R. A. In The Effects of Lunar Dust on Advanced EVA Systems: Lessons from Apollo, Advanced Extravehicular Vehicle Program; NASA, 2005.

[ref32] Banks B. A.; Demko R. In Atomic Oxygen Protection of Materials in Low Earth Orbit; International SAMPE Symposium and Exhibition, 2002; pp 820–832.

[ref33] Sato H.; Shimoyama M.; Kamiya T.; Amari T.; Šašic S.; Ninomiya T.; Siesler H. W.; Ozaki Y. Raman spectra of high-density, low-fensity, and linear low-density polyethylene pellets and prediction of their physical properties by multivariate data analysis. J. Appl. Polym. Sci. 2002, 86, 443–448. 10.1002/app.10999.

[ref34] Shilton S. J.; Prokhorov K. A.; Gordeyev S. A.; Nikolaeva G. Y.; Dunkin I. R.; Smith W. E.; Pashinin P. P. Raman spectroscopic evaluation of molecular orientation in polysulfone. Laser Physics Lett. 2004, 1, 336–339. 10.1002/lapl.200410077.

[ref35] Al-Rubaiai M.; Tsuruta R.; Nam T.; Gandhi U.; Tan X. In Direct Printing of a Flexible Strain Sensor for Distributed Monitoring of Deformation in Inflatable Structures, ASME Conference on Smart Materials, Adaptive Structures and Intelligent System; ASME, 2019.

[ref36] Schaible M. J.; Sjolund K. G.; Ryan E. A.; Shofner M. L.; Reynolds J. R.; Linsey J. S.; Orlando T. M. Performance of chemically modified reduced graphene oxide (CMrGO) in electrodynamic dust shield (EDS) applications 2022. https://arxiv.org/abs/2212.01891.

