# Supplementary material for: Surface-Localized Chemically Modified Reduced Graphene Oxide Nanocomposites as Flexible Conductive Surfaces for Space Applications
Source: ACS Appl Polym Mater. 2023 Jun 29;5(7):5092–102. doi: 10.1021/acsapm.3c00588 (PMC10353001; doi:10.1021/acsapm.3c00588)
Supplement: Supplementary file 1 — ap3c00588_si_001.pdf [file ap3c00588_si_001.pdf]

Supporting Information for “Surface-Localized Chemically Modified Reduced Graphene Oxide Nanocomposites as Flexible Conductive Surfaces for Space Applications”

Emily A. Ryan[a], Zach D. Seibers[b], John R. Reynolds[a,b], and Meisha L. Shofner[a,*]

[a] School of Materials Science and Engineering, Georgia Institute of Technology, Atlanta, Georgia, 30332, United States.
[b] School of Chemistry and Biochemistry, Center for Organic Photonics and Electronics (COPE), Georgia Tech Polymer Network (GTPN), Georgia Institute of Technology, Atlanta, Georgia 30332, United States.

Email: meisha.shofner@mse.gatech.edu

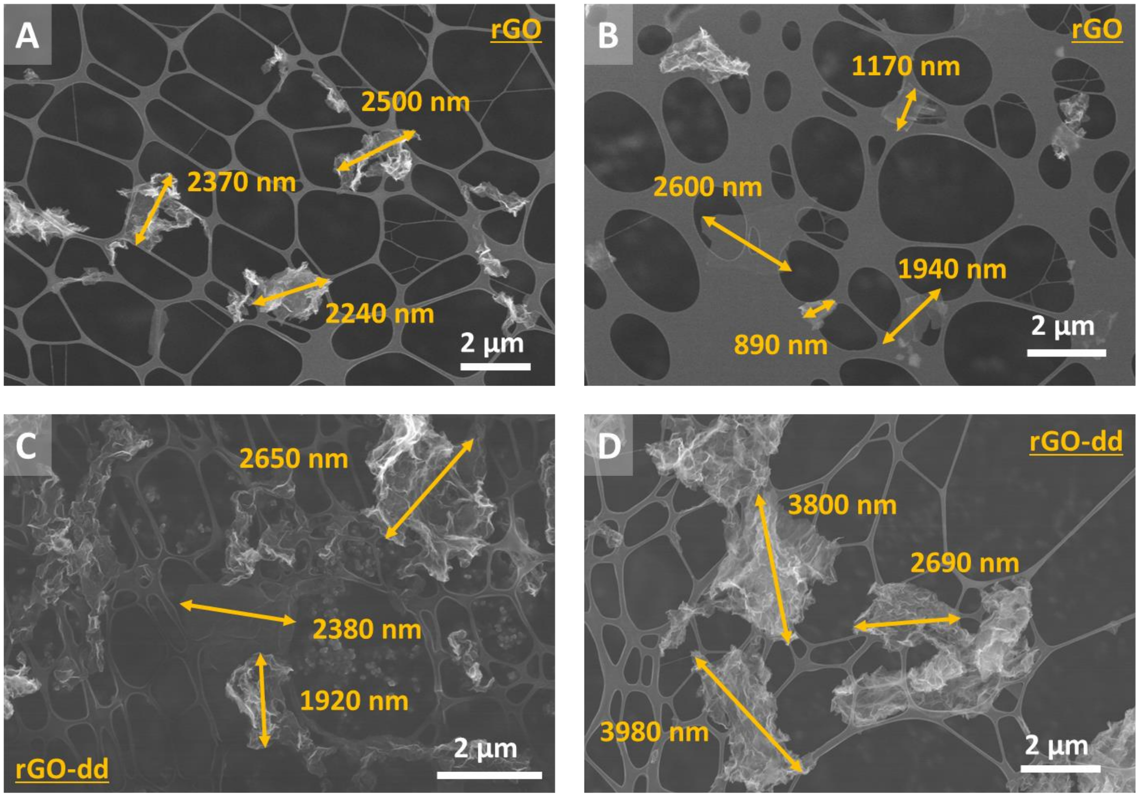

Figure S1. SEM images used for particle size analysis of (A,B) as-received rGO particles and (C,D) functionalized rGO-dd particles.

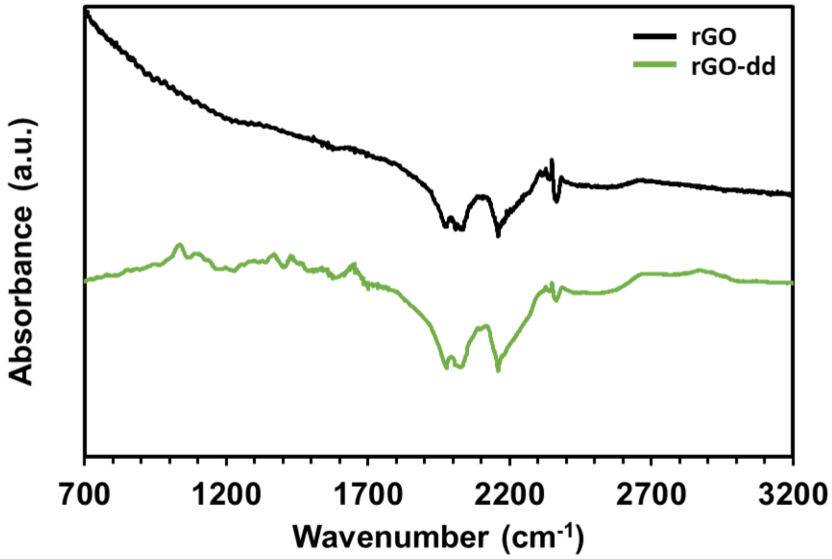

Figure S2. FTIR-ATR spectra of rGO and rGO-dd showing the appearance of new absorbance features between 1300-1500 cm^{-1} commonly associated with methyl rocking modes in alkyl chains indicating successful functionalization with dodecyl groups.

Table S1. Melt infiltration processing temperatures for various materials.

Material	Processing Condition	Target Temperature
HDPE	LT<br>MT<br>HT	112 °C<br>122 °C<br>132 °C
iPP	LT<br>MT<br>HT	145 °C<br>155 °C<br>165 °C
PSU	LT<br>MT<br>HT	195 °C<br>205 °C<br>215 °C

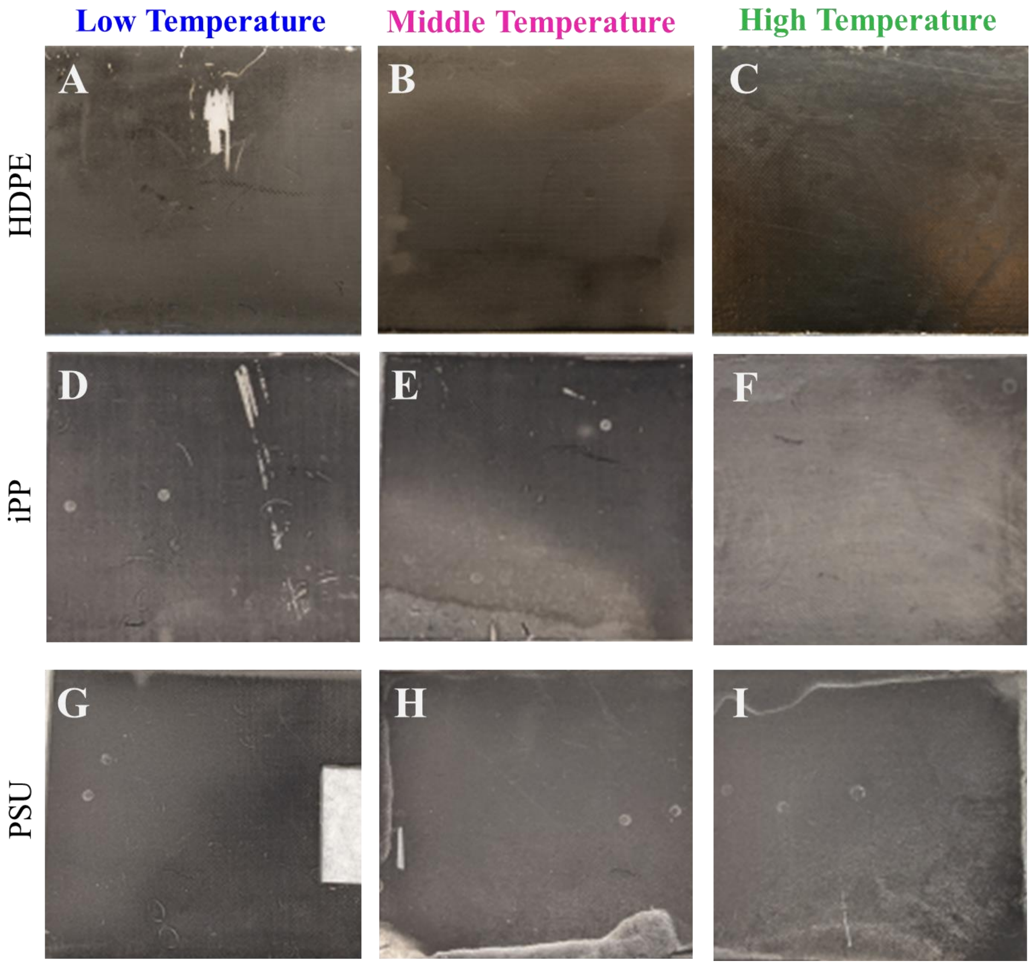

Figure S3. Photographs of nanocomposite surfaces created at three processing temperatures for HDPE, iPP, and PSU substrates. The surfaces become glossier and smoother at higher processing temperatures indicating more complete polymer infiltration.

Surface Profilometry

The LT condition for the HDPE and iPP materials showed patchy, rough surfaces (**Figure S4 A,D**) with mean roughness values (S_a) of 1.21 μm and 1.02 μm, respectively, which appeared matte under visual inspection and is indicative of a randomly oriented particle rich surface. In the MT condition, these substrates showed a reduced S_a of 1 μm and 670 nm, respectively, corresponding with increased visual glossiness and attributed to polymer reaching the surface of the sprayed layer. At this infiltration condition, circular depressions attributed to the surface texture of the mold release film also began to emerge on the HDPE surface, as noted by dashed red circle in **Figure S4 B**. Finally, in the HT condition the surface features of both substrates were dominated by the surface morphology of the mold release film as indicated by the circular indentations, highlighted by the dashed red circles in images of **Figure S4 C,F,** with little contribution from exposed particle roughness indicative of full infiltration. The shallow depth of the indents in the iPP substrates resulted in a further reduction of S_a to 630 nm, while the depth of the features in the HDPE substrates resulted in a slightly increase in S_a to 1.41 μm. The increase in S_a for the highest HDPE processing condition was due to the increased depth of the surface indentations and not increased roughness on the particle scale, as shown by the surface profile plots shown in **Figure S5.**
The PSU substrates followed a similar trend, as seen in **Figure S4 G-I,** with a significant reduction in roughness with increasing processing temperature, from a S_a value of 1.02 μm at the LT condition to 610 nm in the HT condition. Notably, the circular features were less apparent in the PSU substrates. This is attributed to the higher expected infiltrate viscosity of a matrix near its T_g as compared to matrices near their melting regime.

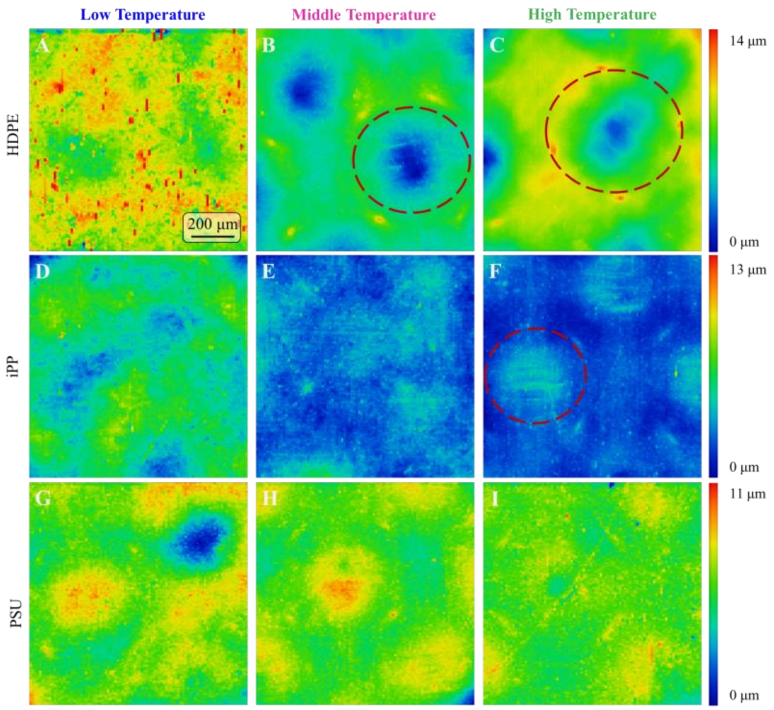

Figure S4. Profilometry of 1mm x 1mm sections from surface localized nanocomposites atop HDPE, iPP, PSU at three temperatures near critical thermal transitions showing reduced surface roughness with increasing processing temperature. Profiles B,C,F include a red dashed circle denoting circular indentation features due to texture of the mold release film. Note: different scales are used for each row of images based on the maximum height measured on a particular substrate.

Table S2. Root mean square (RMS) and mean roughness (S_a) values for each processing condition and substrate measured at a single 1mmx1mm location.

Material	Processing Condition	RMS	S_a
HDPE	Uncoated	1.54 µm	1.23 µm
	LT	1.69 µm	1.21 µm
	MT	1.35 µm	1.00 µm
	HT	1.75 µm	1.41 µm
iPP	Uncoated	1.47 µm	1.31 µm
	LT	1.28 µm	1.02 µm
	MT	840 nm	670 nm
	HT	810 nm	630 nm
PSU	Uncoated	1.11 µm	980 µm
	LT	1.36 µm	1.02 µm
	MT	990 nm	780 nm
	HT	790 nm	610 nm

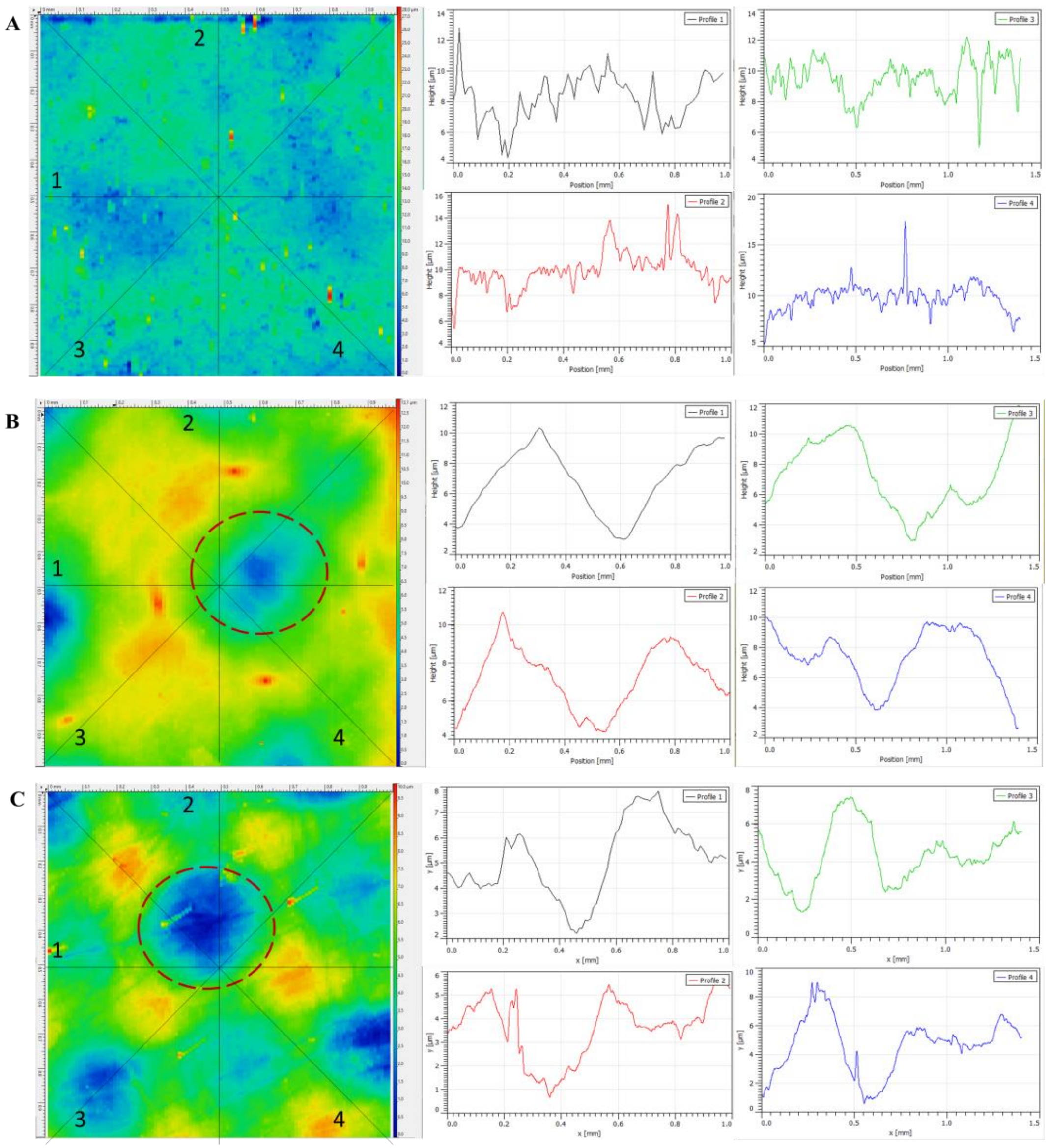

Figure S5. Profilometry images and corresponding line traces of (A) the LT condition HDPE substrate showing significant roughness across the relatively flat surface, (B) the HT condition HDPE substrate showing a relatively smooth profile outside of the deep features from the mold release film, (C) the uncoated side of a HDPE substrate treated at the high processing temperature with the same mold release film showing the same indentation features. Red dashed circles are superimposed to highlight circular indentation regions.

Figure S6 image
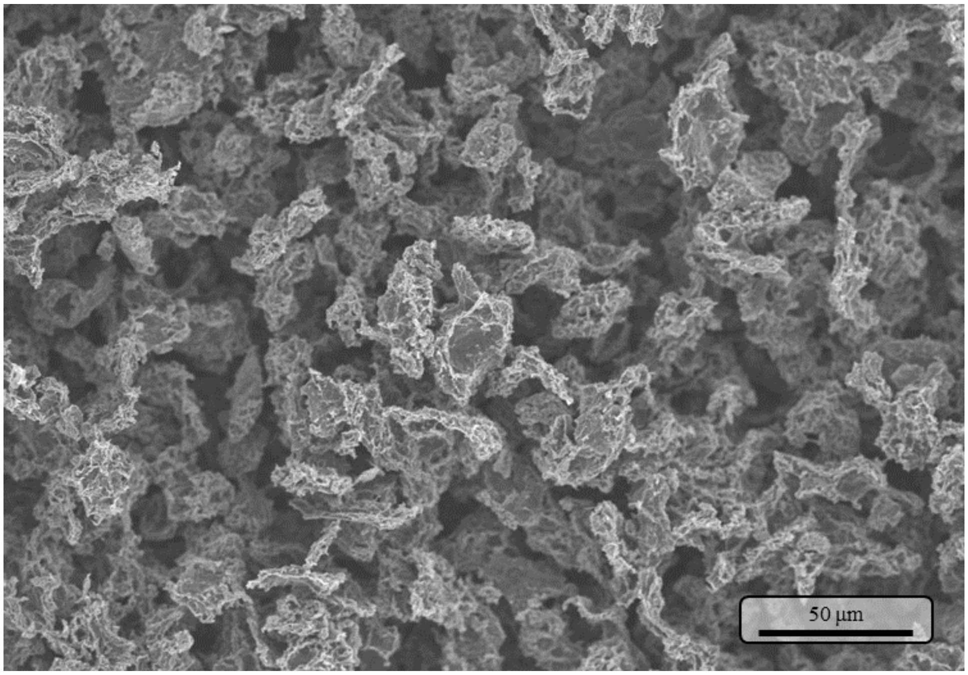

Figure S6. Top-view SEM image of as-sprayed rGO-dd morphology prior to melt pressing showing stacked sheet structures.

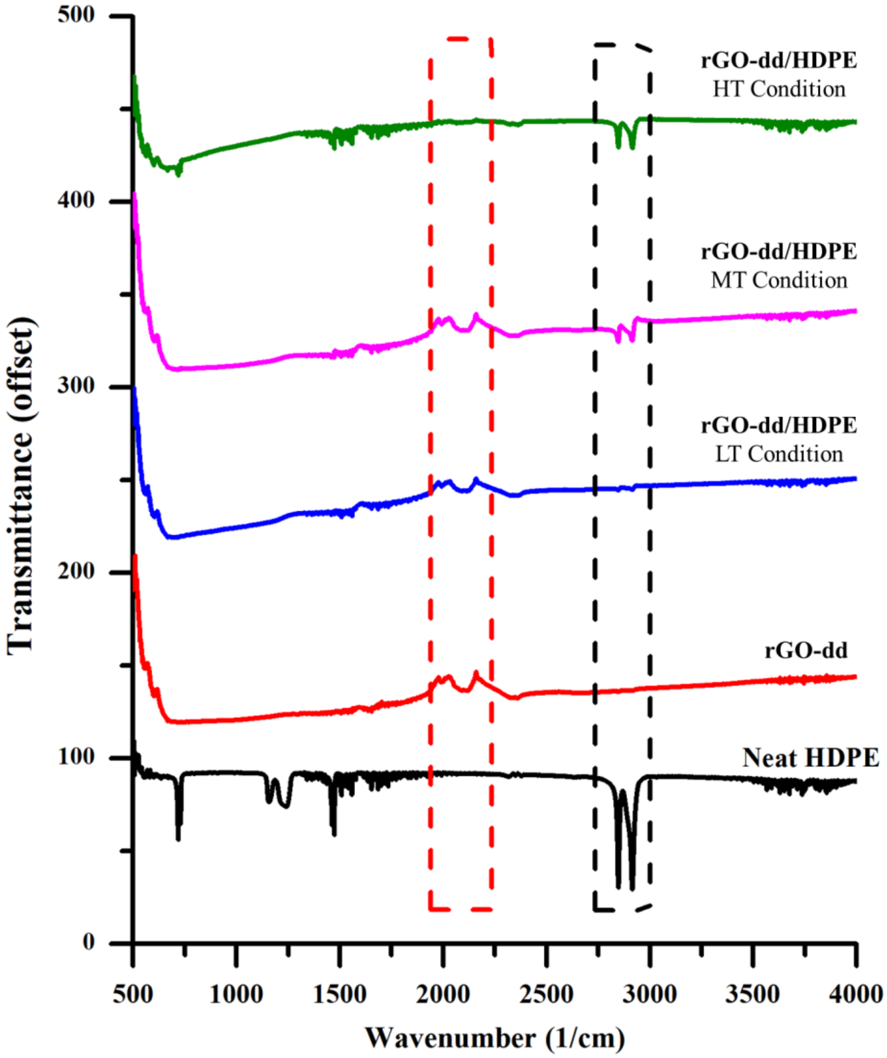

Figure S7. FTIR-ATR spectra of HDPE (black), rGO-dd (red), and surface localized nanocomposites processed at 112 °C (low temperature - blue), 122 °C (middle temperature - pink), and 132 °C (high temperature - green). Signals corresponding to HDPE are increasingly apparent with increasing processing temperature.
